# Development of the Impact of Juvenile Metachromatic Leukodystrophy on Physical Activities scale

**DOI:** 10.1186/s41687-018-0041-x

**Published:** 2018-03-16

**Authors:** T. Michelle Brown, Susan Martin, Sheri E. Fehnel, Linda S. Deal

**Affiliations:** 1 RTI Health Solutions, 200 Park Offices Drive, Research Triangle Park, NC 27709 USA; 2 Pfizer Inc, 500 Arcola Road, Collegeville, 19426 PA USA

**Keywords:** Leukodystrophy, MLD, Metachromatic leukodystrophy, Autosomal recessive enzyme deficiency, Genetic diseases, Patient-reported outcome, Arylsulfatase a deficiency

## Abstract

**Background:**

Metachromatic leukodystrophy (MLD) is a rare disease with three forms based on the age at onset of signs and symptoms. The objective of this study was to develop a caregiver-reported clinical outcome assessment that measures impairments in physical functioning related to activities of daily living in patients with juvenile MLD.

**Methods:**

A targeted literature review and exploration of proprietary research, including a conceptual model, were conducted. Concept elicitation interviews were conducted to elicit additional concepts related to impairments in patients’ physical functioning with caregivers of five individuals with juvenile MLD. Based on the research review and concept elicitation interviews, the conceptual model was updated and the Impact of Juvenile Metachromatic Leukodystrophy on Physical Activities (IMPA) scale draft items were created. Cognitive debriefing interviews were conducted with six additional caregivers to finalize the conceptual model and to refine the IMPA scale.

**Results:**

Initially, 17 potentially important concepts were identified and addressed in the draft IMPA scale. Following the cognitive debriefing interviews, 15 activities/items remained: brush teeth, comb/brush hair, bathe/shower, dress self, eat, drink, use pencil/crayon, sit upright, use toilet, get on/off toilet, walk, use stairs, get in/out of bed, get in/out of chair/wheelchair, and get in/out of vehicle. Items that did not uniquely contribute to the purpose of the instrument were removed.

**Conclusion:**

The IMPA scale, developed according to regulatory standards, provides a means of detecting changes in activities of daily living in individuals with juvenile MLD and can hence be used in future studies to measure benefits of therapeutic interventions.

## Background

Metachromatic leukodystrophy (MLD) is a genetic disorder caused by the deficiency of arylsulfatase A. This deficiency results in the accumulation of sulfatide, which leads to progressive demyelination in the central and peripheral nervous systems, causing various neurological symptoms and early death [1]. MLD is a rare orphan disease with an estimated overall incidence of approximately 1 in 100,000 live births in the Western world [2]. MLD can be classified into three subtypes according to the age of onset: late-infantile MLD (≤ 3 years), juvenile MLD (4-16 years), and adult MLD (> 16 years) [1]. Studies estimate the late-infantile MLD at approximately 30–50% of the MLD cases, followed by the juvenile subtype at approximately 30–40% [3–5].

The natural history of all subtypes of MLD is not well understood, but deterioration of gross motor function is a key feature and progression can be rapid [6]. In a study of 23 patients with late-infantile MLD and 36 patients with juvenile MLD, gait disturbances and abnormal movement patterns were the most frequent first signs in both disease subtypes [7]. In a natural history study that Groeschel and colleagues [8] conducted in 33 children with late-infantile MLD and 35 patients with juvenile MLD, there was more heterogeneity in the juvenile subtype than in the infantile subtype for the following: age at onset, degree of gross motor dysfunction at presentation, and rate of deterioration.

Cognitive decline has been found to accompany or precede gross motor deficits in patients with the juvenile subtype of MLD and may occur in patients with the infantile subtype as well. In a 2010 systematic review of the literature, including 142 studies and 303 cases of MLD, the presenting signs and symptoms of juvenile MLD were both physical and cognitive [9]. These included inattention or difficulties at school (66%), motor or gait abnormalities (26%), tremor or ataxia (18%), neuropathy (13%), and seizures (5%).

Currently, there is no effective treatment or cure for any form of MLD. Hematopoietic stem cell transplant has long been used in juvenile and adult MLD to slow disease progression and to halt cerebral demyelination [10]. This treatment, however, remains controversial given its risks and uncertain long-term effects [11]. Enzyme replacement therapy and gene therapy are new treatments in development for patients with MLD [11].

Owing to limited knowledge of the course of juvenile MLD, its rapid progression, and new potential treatments in development, a brief measure of observable impairments associated with this disorder that are meaningful to patients/parents is needed. Also, owing to the age of individuals with confirmed juvenile MLD, and the severity and rapid progression of observed deficits, any assessment should be completed by primary caregivers, not the patients themselves. The International Society for Pharmacoeconomics and Outcomes Research (ISPOR) Clinician-Reported Outcomes Good Measurement Practices Task Force [12] defines a clinical outcome assessment (COA) as “any assessment that depends on a patient’s volition or a rater’s judgment.” An observer-reported outcome allows caregivers or other observers (not trained healthcare professionals) to assess directly observable concepts in patients who cannot self-report owing to functional limitations (e.g., physical or cognitive deficits). Furthermore, a caregiver-reported COA would be complementary to other clinician-reported, performance-based outcome measures that may be used in clinical trials, such as the functional mobility scale (FMS), gross motor function measure (GMFM-88) and gross motor function classification in MLD (GMFC-MLD) [13–15]. The objective of this study was to develop a new caregiver-reported COA of impairments in physical functioning related to activities of daily living (ADLs) in patients with juvenile MLD.

## Methods

**Fig. 1:**
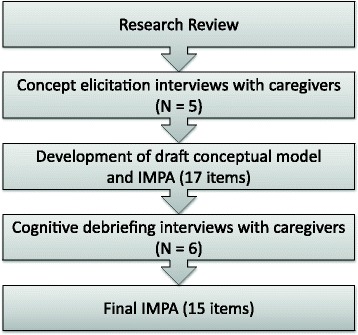
Instrument Development Process. IMPA = Impact of Juvenile Metachromatic Leukodystrophy on Physical Activities

To optimize the measurement properties and ensure the relevance of the new measure to caregivers of individuals with MLD, the instrument development process followed in this study is consistent with the recommendations outlined in the 2009 US Food and Drug Administration (FDA) patient-reported outcomes (PROs) guidance and expectations of European regulators [16, 17]. This study was conducted in accordance with the ethical principles outlined in the Declaration of Helsinki 2008 and reviewed and approved by the RTI International Institutional Review Board. Informed consent was obtained before participation. Figure 1 shows a schematic of the instrument methodological process followed in the development of the content for the new COA measure: Impact of Juvenile Metachromatic Leukodystrophy on Physical Activities (IMPA) scale. The methodological details of each stage of the development process follow.

### Research review

#### Proprietary research

To understand the concepts relevant to the assessment of physical impairments and their impact on ADLs among patients with juvenile MLD, the authors first reviewed the results of proprietary research previously conducted for the study sponsor (data on file with corresponding author). This research review included the following types of reports.

- Literature: representing 33 published peer-reviewed articles, including any relevant instruments, published from January 1, 2002 to December 31, 2012.
- Clinical expert opinion and experience: summarizing seven interviews conducted with MLD clinical experts.
- Caregiver interviews: summarizing seven interviews conducted with caregivers of patients with juvenile MLD, including a preliminary conceptual disease model.

#### Literature

To supplement the previous proprietary research with the most current literature, the authors conducted a new targeted review of the juvenile MLD literature from January 1, 2012 through July 21, 2014. The following terms were searched in PubMed: “metachromatic leukodystrophy, juvenile” or “MLD, juvenile,” yielding 12 references. The terms were cross-referenced to exclude animal research and non-research publication types, and to include only papers in English and with abstracts, resulting in the exclusion of one reference.

### Concept elicitation interviews

To confirm and supplement the research previously conducted, including the proprietary and new additional literature review findings, in-depth concept elicitation interviews were conducted with five caregivers of individuals with juvenile MLD. The objectives of these five interviews were to build upon the information provided during the seven previous caregiver interviews to identify any additional functional impairments associated with the impact of MLD on patients’ ADLs, and to identify appropriate recall periods and potential response scale options to inform development of a draft item pool. The detailed information obtained from these five interviews was anticipated to supplement the existing concept elicitation data to reach the point of concept saturation, and to expand existing knowledge on caregiver assessments of activities of daily living in this rare disease.

Researchers partnered with the MLD Foundation to identify and contact potential qualifying caregivers who could be interviewed at the organization’s annual conference in July 2014. Individuals qualifying for the interviews were 18 years of age and older and caregivers to an individual with juvenile MLD (i.e., onset of MLD at 4-16 years of age). Only one caregiver per child was interviewed. Each 1-h interview was conducted in person by two experienced interviewers using a semi-structured interview guide that incorporated relevant concepts from the research review with a focus on physical impairments associated with MLD, as well as the ways in which these physical deficits limit patients’ abilities to complete ADLs.

Data from the five caregiver interviews were analyzed using a constant comparative analysis paradigm [18]. Dominant trends in the transcripts and field notes from each interview were compared with those of the remaining four interviews to identify patterns in caregivers’ experiences.

### Development of draft conceptual disease model and IMPA scale items

Information from the review of previous proprietary research and concept elicitation interviews informed the new version of the conceptual disease model and the draft items for the IMPA scale. Team members generated draft IMPA scale items following standard instrument development methodologies and best practices to assess only observable physical impairments related to ADLs in patients with juvenile MLD so that caregivers would be able to rate each impairment objectively (i.e., consistent with the development of observer-reported outcomes) [19, 20]. Specifically, the items were designed to address patients’ physical limitations pertaining directly to ADLs that parents/caregivers identified to be important, relevant, and observable.

### Cognitive debriefing interviews

Cognitive debriefing interviews were conducted with six additional caregivers (three iterative pairs of interviews) to pretest and refine the items, as well as to confirm the content of the COA and to test the recall period. The primary goal of the first and second pairs of interviews was to identify potential problems with the wording of the instructions, questions, or response options. The primary goal of the second and third pairs of interviews was to test the adequacy of modifications made to facilitate item understanding and to maximize the accuracy of the response process. Each interview also offered an opportunity to identify any missing physical functioning impacts on ADL issues that participants thought should be addressed, contributing to the evidence for content validity of the final measure.

The MLD Foundation identified and contacted potential qualifying caregivers for the cognitive debriefing interviews, which were conducted in the caregiver’s hometown. The same eligibility criteria as the concept elicitation interviews were utilized for the cognitive debriefing interviews. Each 1.5-h interview was conducted in person by two experienced interviewers using a semi-structured interview guide.

## Results

### Research review

Relevant information from the previous proprietary research was assimilated as new data were collected and results of the current study generated. Signs and symptoms associated with the development of juvenile MLD, their progression, and the various impacts of these physical and non-physical clinical features supported the development of the final IMPA scale and specifically contributed to content of the conceptual disease model.

The new, targeted Medline-indexed, literature review resulted in 11 unique abstracts and four relevant articles selected for full review. Relevant articles related to observable signs and symptoms and treatment outcomes in patients with juvenile MLD. Each of the seven abstracts not selected for review was not relevant either because the study did not specifically pertain to the juvenile subtype of MLD or because the research outcomes did not involve observable neurological functioning.

Supporting the need for COA development, no existing, observer-reported instruments developed to assess physical functioning in patients with MLD were found or referenced in the previous proprietary research or current literature review.

### Concept elicitation interviews

All five caregivers participating in the concept elicitation interviews were parents of children with juvenile MLD. Caregiver and patient characteristics from the concept elicitation interviews are shown in Table 1.

**Table 1:** Caregiver and Patient Characteristics

Characteristic	Concept Elicitation Interviews (*N* = 5)	Cognitive Debriefing Interviews (*N* = 6)
Caregiver		
Sex, female, n (%)	4 (80.0)	3 (50.0)
Age, years, mean (range)	48.6 (37, 58)	55.7 (46, 66)
Race/ethnicity, n (%)		
White	5 (100)	5 (83.3)
Hispanic	–	1 (16.7)
Education level, n (%)		
High school diploma/GED	–	1 (16.7)
Some college	2 (40.0)	3 (50.0)
College degree	3 (60.0)	–
Professional/advanced degree	–	2 (33.3)
Employment status, n (%)		
Full time	1 (20.0)	3 (50.0)
Part time	2 (40.0)	1 (16.7)
Unemployed	2 (40.0)	2 (33.3)
Patient		
Sex, female, n (%)	3 (60.0)	4 (66.7)
Age, years, mean (range)	19.0 (9, 33)	23.2 (13, 33)
Race/ethnicity, n (%)		
White	5 (100)	5 (83.3)
Hispanic	–	1 (16.7)
Age at onset, years, mean	9.4	9.5
median (range)	8.0 (5, 15)	(5, 14) 8.6
Age at diagnosis, years, mean	13.0	10.7
median (range)	14.0 (6, 21)	(3, 14) 11.5
Time between onset and diagnosis, years, mean	3.7	1.1
median (range)	5.0 (0.5, 6.0)	(−5.5, 7.0) 1.1^a^
Time between diagnosis and present, years, mean	6.0	12.5
median (range)	3.0 (1, 19)	(2.0, 21.0) 12.5
Time between onset and present, years, mean	9.6	13.6
median (range)	6.0 (2, 25)	(5.0, 28.0) 13.3

*GED* General Educational Development, *MLD* Metachromatic leukodystrophy

^a^The negative minimum on the range (−5.5) was due to one of the patients receiving a diagnosis before symptom onset because MLD had been diagnosed previously in the child’s older sibling

#### Initial signs and symptoms

Characteristics of the initial signs and symptoms of juvenile MLD, including physical and non-physical concepts, were elicited from the caregivers. Four of the five caregivers indicated that physical, cognitive, and emotional signs and symptoms appeared at approximately the same time; the remaining participant stated that the cognitive and emotional signs and symptoms preceded those physical in nature. The most frequently reported initial physical symptoms included tripping/falling (e.g., “clumsy-like falls”) (*n* = 4), abnormal gait (e.g., foot pronation, knee hyperextension, runs “funny”) (*n* = 3), head/body leaning forward or to the side (*n* = 2), bladder accidents/incontinence (*n* = 2), and swallowing problems (*n* = 2).

#### Progression and impact of signs and symptoms

Across all interviews, caregivers consistently reported on the progression of physical signs and symptoms, the observed impact of these signs and symptoms on patients’ physical functioning, and the need for and types of assistance provided by the caregivers. The physical function concepts and resulting caregiver activities identified as important, relevant, and observable are shown in Table 2.

**Table 2:** Caregiver-Observed Impact of Physical Function on Physical ADLs

Physical Function (Patient cannot)	Physical ADL (Caregiver has to assist with)	Example Caregiver Verbatim
Control bowel and/or bladder	▪ Diapers/pull-ups	▪ *“She wore underwear to school, but you know, I would have more dirty [underwear] than not.”* ▪ *“By the end of grade 2, he was wearing diapers full-time because he was having accidents all the time.”*
Walk and/or stand	▪ Toileting ▪ Walking ▪ Standing ▪ Bathing/showering ▪ Moving/transfers	▪ *“She walked with a walker for a very short time. So between 3, 4 months later, she quit [walking] altogether.”* ▪ *“He leaned more and more … I guess he was developing weakness in his legs.”* ▪ *“Physically, she was having trouble stepping over the bathtub to get into the bathtub. So she fell a couple times.”* ▪ *“We took a summer vacation…and we kept pulling off and stopping to get out and look at the scenery. He wouldn’t get out of the car. Well, he was having trouble getting out of the car, but he didn’t tell us.”*
Go up and down stairs	▪ Going up/down stairs	▪ *“Stumbling on stairs — and it’s usually going up the stairs. And she used to joke, ‘Oh, I’m being blonde. I’m tripping up [the] stairs. Ha, ha, ha.’ It was always a joke. But now I notice it at least five times a week.”*
Swallow/swallow without choking	▪ Eating ▪ Drinking	▪ *“For the 6 months prior to [getting the feeding tube], we were slowly seeing a decline … we went from his eating regular foods to purees.”* ▪ *“She started choking on liquids because she couldn’t swallow very well.”* ▪ *“I always make sure he’s sitting straight up at a 90-degree angle, and I also make sure that he’s talking a little bit after he sucks on the straw, so that sort of helps him to swallow better.”*
Use hand to reach, grab, or hold an object	▪ Feeding ▪ Brushing teeth/hair ▪ Dressing ▪ Bathing/showering ▪ Writing	▪ *“She has a really tight grip. So she can self-feed a little bit for some things, some finger foods …”* ▪ *“It was easier for him to hold a cup before on his own, but that’s kind of hard to do a little bit now. And when he grasps something, sometimes it’s just hard for him to let go. He has a really tight grip.”* ▪ *“She would pick stuff up or like try to help and get maybe a couple feet and … she wouldn’t have the grip or strength. And she would drop it.”* ▪ *“He’s clumsy putting on long pants. He’s still independent doing it. It just takes him longer.”* ▪ *“Her handwriting started getting really small. I noticed she was having trouble holding the pen.”*
Control head and trunk	▪ Sitting	▪ *“Then in grade 2, he lost upper body control, he started falling over in the booster seat. So we’d go around a turn and he’d fall over. So, we had to put him back into a car seat.”* ▪ *“Like if I leave her [sitting] on the couch and I’m not there, she goes this way, she’s going down. She’s not getting back up.”*

*ADLs* Activities of daily living, *MLD* Metachromatic leukodystrophy

To protect the identity of participants and individuals with juvenile MLD, quotations were arbitrarily revised to represent male or female patients

At the time of the interviews, the mean time between the onset of signs and symptoms and the study was 9.6 years (range, 2-25 years). The progression of functional limitations was still apparent for some patients but had slowed or plateaued for others. While the presenting signs and symptoms and the speed of decline varied among patients, the progression of functional limitations followed similar patterns across patients.

When asked about the length of time in which patients tended to progress, such that the caregivers would be able to observe a difference in function, most caregivers referenced weeks or months. They reported that the speed of change varied over the course of the disease, but rarely, if ever, did it change from day to day. Caregivers agreed that the report of observable changes over the past 7 days would be appropriate.

### Development of draft IMPA items

Nine ADL categories and 17 physical activity concepts were identified from the concept elicitation interviews and supported by information obtained from the previous proprietary research and literature review. Items were selected to assess observable physical limitations generally related to standard ADL categories, such as grooming, bathing, dressing, eating/drinking, writing/drawing, sitting, toileting, mobility, and transfers. Unobservable (or inconsistently observable) and non-physical behaviors related to other instrumental ADL categories (i.e., communication, social/family roles, school attendance/activities) were not included. The 17 basic ADL concepts formed the 17 items tested in the first pair of cognitive debriefing interviews (Table 3).

**Table 3:** Caregiver-Observed Activities by ADL Category

Caregiver-Observed Physical ADLs	
Grooming	
1. Brush teeth	
2. Comb/brush hair	
Bathing	
3. Take a bath/shower	
Dressing	
4. Dress self	
Eating/drinking	
5. Use a feeding tube^a^	
6. Eat	
7. Drink	
Writing/drawing	
8. Use pencil/crayon	
Sitting	
9. Sit upright	
Toileting	
10. Get on/off toilet^b^	
Mobility	
11. Use a wheelchair^a^	
12. Walk inside the home^c^	
13. Walk outside the home^c^	
14. Use stairs	
Transfers	
15. Get in/out of bed	
16. Get in/out of a chair/wheelchair	
17. Get in/out of a vehicle	

*ADL* Activities of daily living, *IMPA* Impact of Juvenile Metachromatic Leukodystrophy on Physical Activities

^a^Item removed from the final IMPA scale

^b^An additional item, “use the toilet,” was added to the final IMPA scale

^c^Items relating to walking “inside” and “outside” the home were combined into a general item related to walking in the final IMPA scale

### Cognitive debriefing interviews

All six caregivers who participated in cognitive debriefing interviews were parents of children with juvenile MLD; caregiver and patient characteristics are shown in Table 1.

To improve consistency in respondent interpretation, modifications were made to individual items. For example, after review from the first pair of interviews, three items in the eating/drinking category were consolidated into two items: eating food and drinking liquids; and four items in the ADL mobility category were reduced to two: walking and using stairs. In addition, one item was added to the toileting ADL category (use the toilet). A total of 15 items/concepts and 9 ADL categories were tested in the remaining two pairs of interviews. All of these items and categories were retained in the final instrument.

Modifications were also made to the item response scales. Two response scales were tested in the first pair of interviews: the first was based on levels of assistance (i.e., “with no assistance,” “with a little assistance,” “with a lot of assistance,” and “could not do”), and the second was based on evaluated difficulty (i.e., “on his/her own [with no assistance],” “with some difficulty [needed a little assistance],” “with great difficulty [needed a lot of assistance],” and “could not do”). The first response scale was generally clearer and easier for caregivers to use. Some response options were also refined and modified for the final IMPA scale. For example, one iteration provided additional specificity and clarity by naming the physical activity in the item: “could not do” became “could not walk (completely unable to support body or assist with movement)” or “could not do (completely unable to support body or assist with movement).”

Caregivers reported that it was easy for them to recall and answer questions pertaining to their children’s ADLs over the past 7 days, indicating little to no variability in physical functioning, even during the onset of functional losses. Caregivers confirmed the items presented in the IMPA scale addressed the relevant and important functional limitations significant to them and their children. No new concepts related to the impact of physical function deficits were introduced by the six participants of the cognitive debriefing interviews, beyond those elicited in the concept elicitation interviews, demonstrating saturation of information (i.e., the point at which no new information is being gleaned from the qualitative research). Furthermore, no participant identified any performance concept of ADLs in patients with MLD as missing from the draft IMPA scale.

### Final IMPA scale and conceptual disease model

**Fig. 2:**
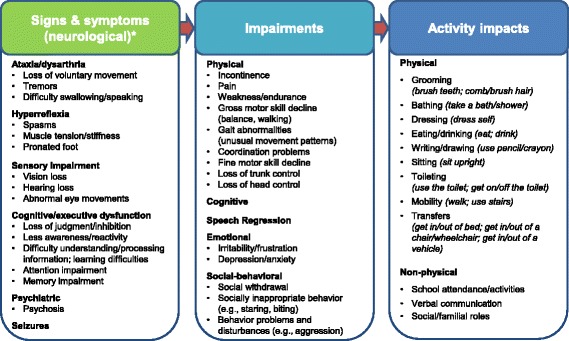
Juvenile metachromatic leukodystrophy conceptual disease model. Content in italics denote the 15 concepts represented in the IMPA scale. While this model depicts both physical and nonphysical functioning effects and ADL impacts, the goal of the IMPA scale was to measure observable impairments in physical functioning. *This model was based on an early model developed in the previous research and modified based on the research conducted in the current study. IMPA = Impact of Juvenile Metachromatic Leukodystrophy on Physical Activities

The final conceptual disease model incorporated information from all steps in the instrument development process. The model and content of the IMPA scale (as shown in italics in the “Activity impacts” box) is shown in Fig. 2. The final set of 15 ADLs includes: brush teeth, comb/brush hair, take a bath/shower, dress self, eat, drink, use pencil/crayon, sit upright, use the toilet, get on/off the toilet, walk, use stairs, get in/out of bed, get in/out of a chair/wheelchair, and get in/out of a vehicle.

## Discussion

Taken together, the results of the previous research, supplemental literature review, and caregiver input strongly supported the development of a questionnaire specifically designed to measure behaviors directly observable by caregivers of individuals with juvenile MLD. The conceptual disease model and associated IMPA scale provide critical tools in the study of this disease. This 15-item IMPA scale is the first reported tool to measure the impact of physical impairments on basic ADLs in individuals with juvenile MLD (i.e., grooming, bathing, dressing, eating/drinking, writing/drawing, sitting, toileting, mobility, and transfers). Additionally, as an observer-reported outcome measure, the IMPA scale was rigorously developed to be consistent with the FDA’s guidance on the use of PROs in product labeling [16], supporting its use in future clinical trials in patients with juvenile MLD.

Furthermore, information from the previous proprietary and current research significantly contributes to the understanding of MLD and the knowledge base of disease signs and symptoms, as well as its associated impairments and activity limitations. To our knowledge, this conceptual disease model of juvenile MLD is the first disease model specific to MLD or juvenile MLD. This model links the signs and symptoms of juvenile MLD to specific limitations and functional activities, thereby depicting the direct impact of disease on the daily lives of patients with juvenile MLD and of their caregivers. Importantly, the comprehensive conceptual disease model developed in this study depicts both physical and non-physical functional impacts of the disease, despite the intentional focus of the IMPA scale on observable decrements in the ability to complete ADLs, skills essential in preserving personal wellbeing, development, and age-appropriate levels of autonomy.

Limitations of the current study involve its generally small sample size (11 caregiver interviews); however, because juvenile MLD is a rare condition, we believe that this sample size is reasonable. And while the number of interviews was small, these interviews were targeted (i.e., physical impacts/limitations on ADLs) towards building upon information already obtained from the previous proprietary research (including seven caregiver interviews). Additionally, and most importantly, the results of this research were consistent with the previous caregiver interviews and literature in identifying signs of MLD such as gait disturbance, cognitive decline, difficulties in school, and incontinence [6, 7, 21].

Two additional limitations were also related to the rarity of MLD. One was the long recall period which caregivers referenced when describing the onset and progression of their children’s disease and its impact on physical functioning. While four of the 11 patients cared for by interview participants had received diagnoses within the past 3 years, caregivers of patients who had received diagnoses many years ago (up to 21 years ago) were also accepted for research participation given the rarity of the disease and recruiting challenges. Long recall periods such as these could introduce bias or error because caregivers have to rely extensively on their memories. However, because of the consistent input from caregivers and demonstration of saturation across the 11 caregiver interviews, a bias related to the long reference period is unlikely. In particular, no new information specific to the impact of physical function decline associated with juvenile MLD on ADLs was introduced during the five concept elicitation interviews; this was further supported when no new concepts were provided by the six cognitive debriefing interview participants. As such, these factors provide evidence that the qualitative research population was sufficient and valid for the purposes of developing the IMPA scale. Another limitation was a potential selection bias due to recruitment through the MLD Foundation, as it is possible that caregivers that are part of this patient advocacy organization may differ in some respects to those who are not.

While the IMPA scale was developed using standard and rigorous methods, additional research is needed to confirm and support this measure fully. Most importantly, finalization of the IMPA scale will depend on a future study for psychometric evaluation of the measure, involving comparison of the IMPA scale with other accepted measures of function (e.g., cognitive, behavioral, ADLs). Specifically, the optimal structure and scoring algorithm need to be determined, and the measurement properties (reliability, validity, and ability to detect change) of the scale need to be assessed and documented.

## Conclusion

The IMPA scale was developed in accordance with the FDA PROs guidance to measure the impact on ADLs attributable to physical impairments in individuals with juvenile MLD. It is a brief, content-valid scale designed to be completed by caregivers as appropriate patient surrogate responders based on their direct observations. Once comprehensive psychometric evaluation of the IMPA scale addresses the remaining requirements, the IMPA scale is expected to facilitate the evaluation of meaningful treatment benefit in clinical trials of new treatments for patients with juvenile MLD.

## Abbreviations

ADL: Activity of daily living
COA: Clinical outcome assessment
FDA: Food and Drug Administration
IMPA: Impact of Juvenile Metachromatic Leukodystrophy on Physical Activities
MLD: Metachromatic leukodystrophy
PRO: Patient-reported outcome
